# Unilateral versus bilateral pedicle screw fixation with posterior lumbar interbody fusion for lumbar degenerative diseases: A meta-analysis

**DOI:** 10.1097/MD.0000000000006882

**Published:** 2017-05-26

**Authors:** Huan Liu, Ying Xu, Si-Dong Yang, Tao Wang, Hui Wang, Feng-Yu Liu, Wen-Yuan Ding

**Affiliations:** a Department of Spine Surgery, The Third Hospital of Hebei Medical University; b Department of Cardiology, The Traditional Chinese Medicine Hospital of Hebei Medical University, Shijiazhuang, Hebei, P.R. China.

**Keywords:** bilateral fixation, lumbar degenerative diseases, meta-analysis, posterior lumbar interbody fusion, unilateral fixation

## Abstract

**Background:**

Both unilateral pedicle screw fixation with posterior lumbar interbody fusion (PLIF) and bilateral pedicle screw fixation with PLIF are used to treat lumbar degenerative diseases (LDD). However, which one is a better treatment for LDD remains considerable controversy. Therefore, the focus of this meta-analysis was to assess the merits and shortcomings of efficacy of these 2 surgical procedures for LDD.

**Methods:**

An extensive search of literature was performed in Pubmed/MEDLINE, Embase, CNKI, and WANFANG databases on unilateral versus bilateral pedicle screw fixation with PLIF fusion for LDD, from January 2007 to January 2017 and language was restricted to Chinese or English. The following variables were extracted: blood loss, operation time, length of hospital stay, Japanese Orthopedic Association (JOA) scores, visual analog scale (VAS) and Oswestry disability index (ODI) scores, fusion rate, total complications, infection, dural injury, and nerve injury. Data analysis was conducted with RevMan 5.3 and STATA 12.0.

**Results:**

A total of 11 studies containing 844 patients were included in our study. The results showed that unilateral is better than bilateral pedicle screw fixation with PLIF in blood loss (*P* < .00001), operation time (*P* < .00001), the length of hospital stay (*P* = .003), and the final follow-up ODI scores (*P* = .04). However, there are no significant differences in JOA, VAS, and preoperative ODI scores. There are also no significant differences in fusion rate and complications (all *P* > .05).

**Conclusion:**

Based on our meta-analysis, our results suggest that both unilateral pedicle screw fixation with PLIF and bilateral pedicle screw fixation with PLIF for LDD have effective results in clinical outcomes. Both 2 methods may result in clinical improvement and similar outcomes of fusion rate and complications; However, compared with bilateral fixation, unilateral fixation produces more satisfactory efficacy in the blood loss and the operation time.

## 1 Introduction

Lumbar degenerative diseases (LDD) is a common disease which including prolapsed lumbar intervertebral disc, degenerative instability, spondylolisthesis of lumbar, lumbar spinal stenosis, and degenerative scoliosis. Posterior lumbar interbody fusion (PLIF) has gradually become a common and effective surgical method to treat LDD, since first reported by Dr Ralph Cloward more than 60 years ago.^[1]^ PLIF could achieve a decompression of the dura sac and nerve roots, maintain proper disk space height, and accelerate the recovery process from spine surgery.^[2–4]^

Traditionally, bilateral pedicle screw fixation was widely used for managing PLIF, which could provides promote arthrodesis, prevent nonunion, and improve fusion rate.^[5]^ However, due to the excessive rigidity of bilateral fixation, this procedure has also been suspected to result in more extensive dissection, adjacent segments degeneration, device-related osteoporosis, and higher risk of implant-related complications.^[6–8]^ Also, it may mean more extensive dissection, more blood loss, longer duration of operation, and greater medical costs. In recent years, unilateral pedicle screw fixation in PLIF was used to decrease the stiffness of the instrumented segment, and acquired similar functional results and fusion rate compared with bilateral fixation in the management of LDD.^[9–14]^

Previous meta-analyses^[15–17]^ reviewed mainly focused on the comparison between bilateral and unilateral pedicle screw fixation with transforaminal lumbar interbody fusion (TLIF) or minimally invasive surgery TLIF, with few variables or included studies. The clinical efficacy and complications of unilateral compared with bilateral screw fixation with PLIF for LDD still remain controversial. Thereby, we conduct this meta-analysis to critically assess the effectiveness and safety of these 2 techniques for the treatment of LDD.

## 2 Materials and methods

### 2.1 Ethics statement

There is no need to seek consent from patients, as in this study all the data were collected from the published data and analyzed anonymously without any potential harm to the patients; this study was approved by the Ethics Committee of our hospital.

### 2.2 Search strategy

An extensive search of literature was performed in PubMed, Embase, the Cochrane library, CNKI, and WANFANG databases published from January 2007 to January 2017. The search was conducted with the use of the following search terms: “unilateral pedicle screw fixation,” “bilatera pedicle screw fixation,” “posterior lumbar interbody fusion,” and “lumbar degenerative diseases,” with various combinations of the operators “AND” and “OR.” Language was restricted to Chinese and English.

### 2.3 Inclusion criteria

Studies were included if they met the following criteria: age between 30 and 75 years; included patients with LDD, such as degenerative lumbar disk herniation, lumbar spinal stenosis, and degenerative spondylolisthesis; included patients who underwent PLIF; and unilateral and bilateral pedicle screw fixation were compared.

### 2.4 Exclusion criteria

Studies were excluded if they met the following criteria: had an average follow-up time of less than 6 months; patients with spinal deformity, trauma, spinal tumor, or with previous lumbar operation; only described unilateral or bilateral screw fixation; without PLIF; involved patients with another disease, such as severe osteoporosis, active infection, cervical spondylosis, or thoracic disease; in vitro human cadaveric biomechanical studies; and earlier trials, reviews, and case-reports.

### 2.5 Selection of studies

All subjects, abstracts, and the full text of articles were independently reviewed by 2 reviewers. Then the eligible trials were selected according to the inclusion criteria. If they disagreed, a 3rd reviewer was consulted to resolve the differences.

### 2.6 Data extraction and management

Two reviewers extracted data independently. The extracted information included: author; year of publication; the country of study, study type, the sample size, the mean age of participants, gender, and duration of follow-up, blood loss, operation time, the clinical outcomes (Japanese Orthopedic Association [JOA], Oswestry disability index [ODI], visual analog scale [VAS] score, and the length of hospital stay), fusion rate, and complications.

### 2.7 Statistical analysis

All data analyses were performed using RevMan 5.3 and STATA 12.0. For dichotomous variables, we analyzed using odds ratio (OR), and for continuous variables, the standardized mean difference (SMD) was used. Both were reported with 95% confidence intervals, and the heterogeneity test was considered statistically significant when *P* < .05. We used *I*^2^ statistic to assess heterogeneity. *I*^2^ > 50% implied substantial heterogeneity among the included studies, random-effect model was used to analysis. If *I*^2^ less than or equal to 50%, which were considered to represent no significant heterogeneity, we chose fixed-effect model.

### 2.8 Test for risk of publication bias

Funnel plot was performed to evaluate the risk of publication bias. If the funnel plot was asymmetric, there is publication bias and symmetric indicated no publication bias. The funnel plot asymmetry was measured by Begg and Egger tests. *P* values less than .05 were regarded as a significance level.

## 3 Results

### 3.1 Search results

**Figure 1:**
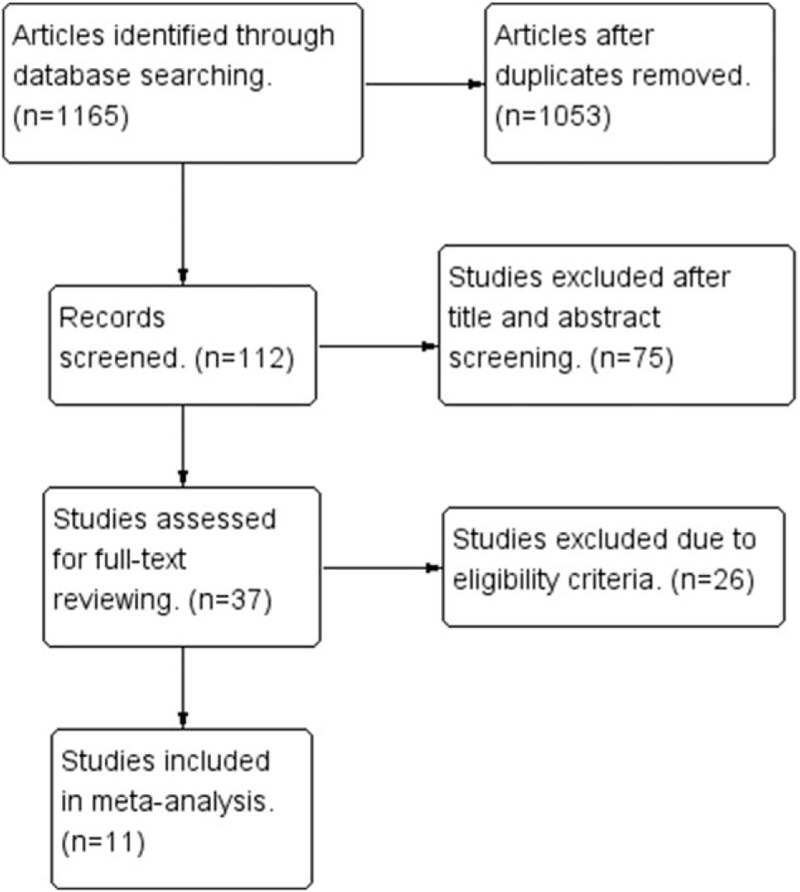
Flow diagram of study selection.

Through the application of search strategy, a total of 504 English studies in Pubmed/MEDLINE and Embase, 661 Chinese studies in CNKI and WANFANG were identified. Of these, 1053 articles after duplicates were removed; 75 articles were excluded after title and abstract screening. Twenty-six articles were excluded due to eligibility criteria. As a result, a total of 11 studies^[3,9,18–26]^ were identified for this meta-analysis, including 2 English article and 9 Chinese articles. The flow diagram of the study search process is presented in Fig. 1.

### 3.2 Baseline characteristics and quality assessment

A total of 11 studies comprising 398 patients treated with unilateral and 446 patients treated with bilateral screw fixation with PLIF, respectively, were included in the final analysis. There were no significant differences between groups regards to age, sex, and follow-up (Table 1), presents the baseline characteristics of the 2 groups. To assess the quality of each study, we used the Newcastle Ottawa Quality Assessment Scale (NOQAS). This scale for nonrandomized case controlled studies and cohort studies had a maximum of 9 points, which included the quality of selection, comparability, exposure, and outcomes for study participants. Of these studies, 8 scored 8 points and 3 scored 7 points. Therefore, the quality of each study was relatively high (Table 2).

**Table 1:**
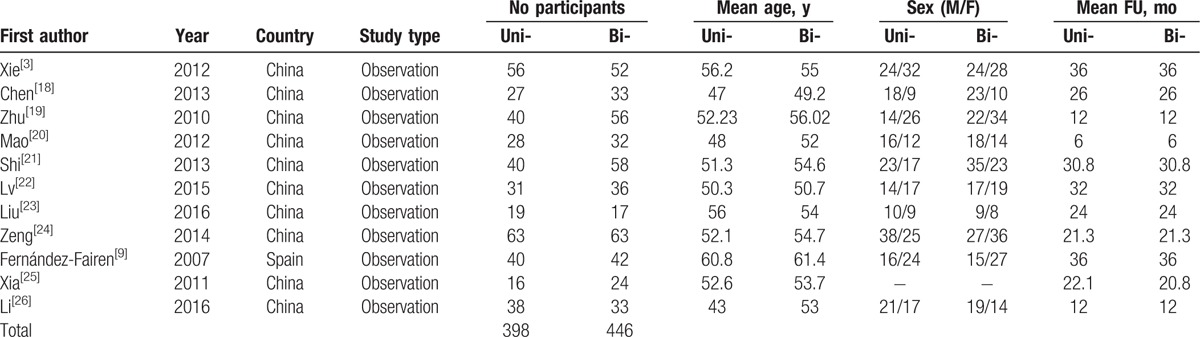
Characteristics of included studies.

**Table 2:**
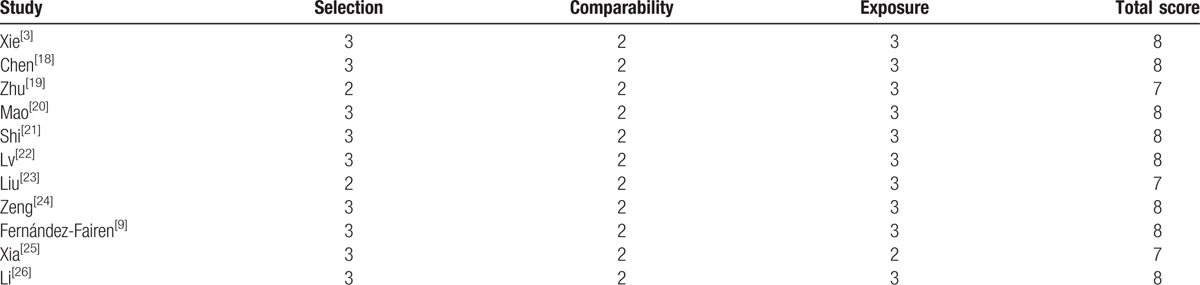
The quality assessment according to the Newcastle Ottawa Quality Assessment Scale (NOQAS) of each study.

### 3.3 Blood loss and operation time

**Figure 2:**
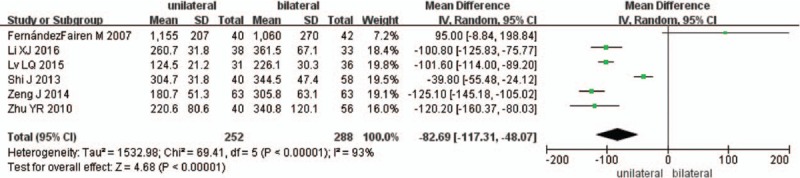
The standardized mean difference (SMD) estimate for blood loss.

Six studies reported the intraoperative blood loss between unilateral and bilateral pedicle screw fixation with PLIF (n = 252 in the unilateral group, and 288 in the bilateral group). Meta-analysis showed unilateral group had less blood loss than bilateral group (*P* < .00001, SMD = −82.69 [−117.31, −48.07]; heterogeneity: *P* < .00001, *I*^2^ = 93%, random-effect model, Fig. 2).

**Figure 3:**
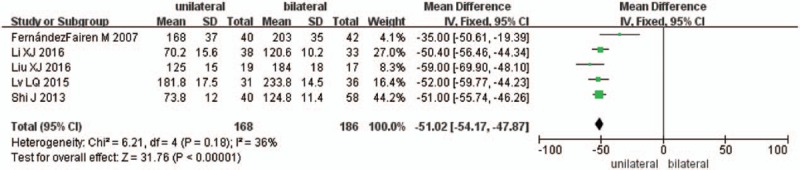
The standardized mean difference (SMD) estimate for operation time.

Five studies reported the operation time (n = 168 in the unilateral group, and 186 in the bilateral group). The unilateral group showed shorter operation time compared with the bilateral group (*P* < .00001, SMD = −51.02 [−54.17, −47.87]; heterogeneity: *P* < .18, *I*^2^ = 36%, fixed-effect model, Fig. 3).

### 3.4 Clinical outcomes

**Figure 4:**
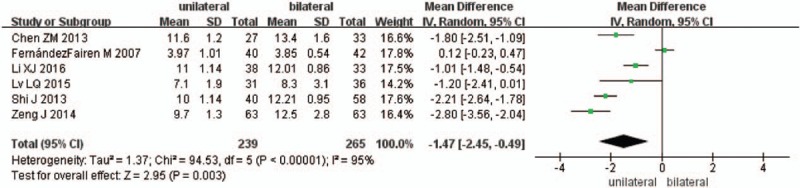
The standardized mean difference (SMD) estimate for length of hospital stay.

Six studies reported the length of hospital stay between unilateral and bilateral pedicle screw fixation with PLIF (n = 239 in the unilateral group, and 265 in the bilateral group). Meta-analysis showed unilateral group had shorter hospital stay than bilateral group. (*P* = .003, SMD = −1.14 [−2.45, −0.49]; heterogeneity: *P* < .00001, *I*^2^ = 95%, random-effect model, Fig. 4).

**Figure 5:**
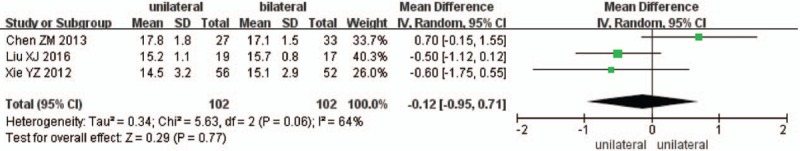
The SMD estimate for preoperative JOA scores. JOA = Japanese Orthopedic Association, SMD = standardized mean difference.

**Figure 6:**
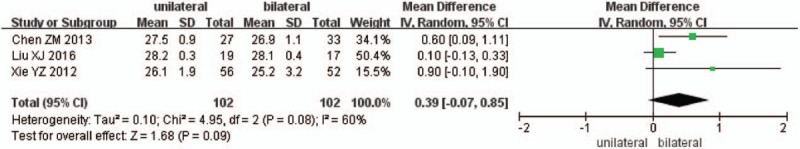
The SMD estimate for the final follow-up JOA scores. JOA = Japanese Orthopedic Association, SMD = standardized mean difference.

Three studies reported the preoperative and the final follow-up JOA scores (n = 102 in the unilateral group, and 102 in the bilateral group). There is also no significant difference between the 2 groups in preoperative JOA scores (*P* = .77, SMD = −0.12 [−0.95, −0.71]; heterogeneity: *P* = .06, *I*^2^ = 64%, random-effect model, Fig. 5), but significant difference in the final follow-up JOA scores (*P* = .09, SMD = −0.39 [−0.07, 0.85]; heterogeneity: *P* = .08, *I*^2^ = 60%, random-effect model, Fig. 6).

**Figure 7:**
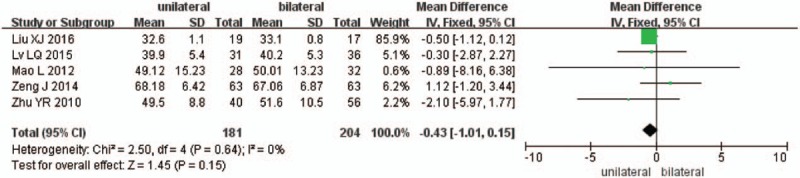
The SMD estimate for preoperative ODI scores. ODI = Oswestry disability index, SMD = standardized mean difference.

**Figure 8:**
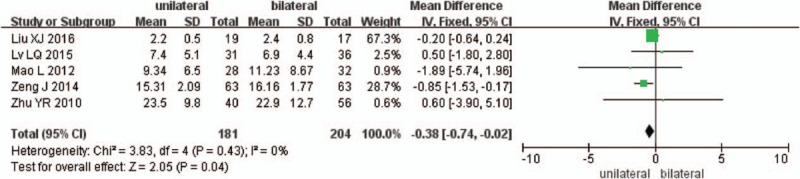
The SMD estimate for the final follow-up ODI scores. ODI = Oswestry disability index, SMD = standardized mean difference.

Five studies reported the preoperative and the final follow-up ODI scores (n = 181 in the unilateral group, and 204 in the bilateral group). There is no significant difference between the 2 groups in preoperative ODI scores (*P* = .15, SMD = −0.43 [−1.01, 0.15]; heterogeneity: *P* = .64, *I*^2^ = 0%, fixed-effect model, Fig. 7), but significant difference in the final follow-up ODI scores (*P* = .04, SMD = −0.38 [−0.74, −0.02]; heterogeneity: *P* = .43, *I*^2^ = 0%, fixed-effect model, Fig. 8).

**Figure 9:**
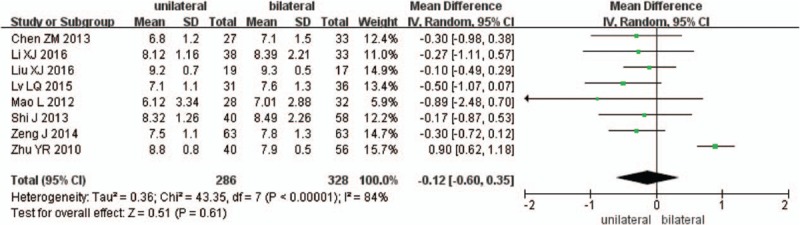
The SMD estimate for preoperative VAS scores. SMD = standardized mean difference, VAS = visual analog scale.

**Figure 10:**
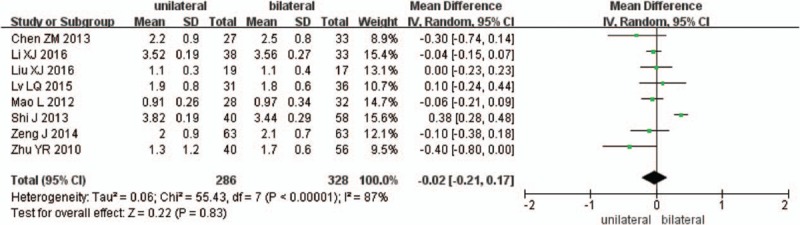
The SMD estimate for the final follow-up VAS scores. SMD = standardized mean difference, VAS = visual analog scale.

Eight studies reported preoperative and the final follow-up VAS scores (n = 286 in the unilateral group, and 328 in the bilateral group). Preoperative VAS scores were similar between the 2 groups (*P* = .61, SMD = −0.12 [−0.6, 0.35]; heterogeneity: *P* < .00001, *I*^2^ = 84%, random-effect model, Fig. 9), the final follow-up VAS scores were also similar between the 2 groups (*P* = .83, SMD = −0.02 [−0.21, 0.17]; heterogeneity: *P* < .00001, *I*^2^ = 87%, random-effect model, Fig. 10).

### 3.5 Fusion rate

**Figure 11:**
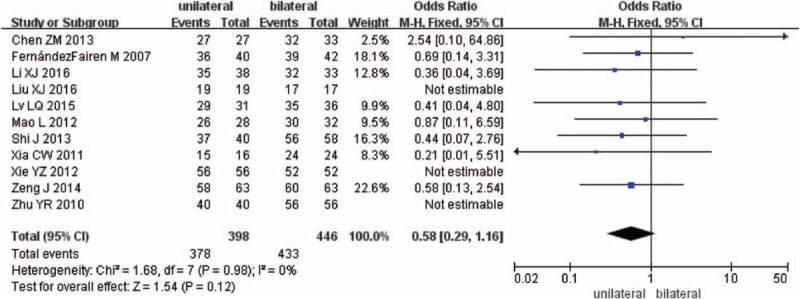
The standardized mean odds ratio (OR) estimate for fusion rate.

Eleven studies reported fusion rate between unilateral and bilateral pedicle screw fixation with PLIF (n = 398 in the unilateral group, and 446 in the bilateral group). The meta-analysis showed that unilateral has a similar result of fusion rate compared with bilateral (*P* = .12, OR = 0.58 [0.29, 1.16]; heterogeneity: *P* = .98, *I*^2^ = 0%, fixed-effects model Fig. 11)

### 3.6 Complications

**Figure 12:**
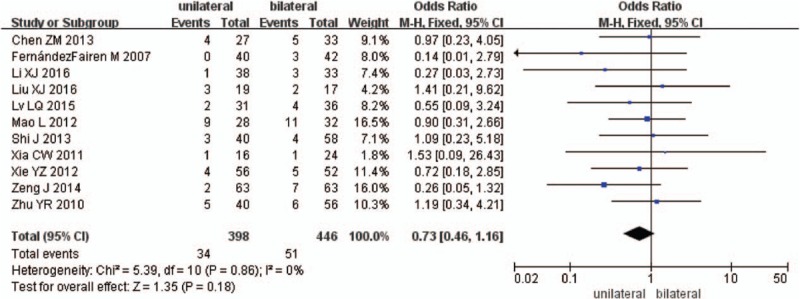
The standardized mean odds ratio (OR) estimate for total complications.

Eleven studies reported total complications between unilateral and bilateral pedicle screw fixation with PLIF (n = 398 in the unilateral group, and 446 in the bilateral group). Total complications were similar between the 2 groups (*P* = .18, OR = 0.73 [0.46, 1.16]; heterogeneity: *P* = .86, *I*^2^ = 0%, fixed-effects model, Fig. 12).

**Figure 13:**
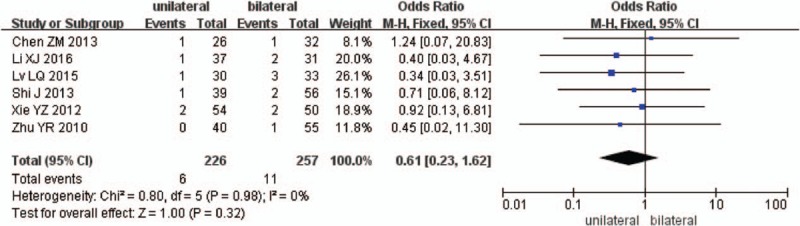
The standardized mean odds ratio (OR) estimate for infection.

Six studies reported the infection after surgery between unilateral and bilateral pedicle screw fixation with PLIF (n = 232 in the unilateral group, and 268 in the bilateral group). Meta-analysis showed unilateral group was similar compared with bilateral group in infection. (*P* = .32, OR = 0.63 [0.23, 1.62]; heterogeneity: *P* = .98, *I*^2^ = 0%, fixed-effect model, Fig. 13).

**Figure 14:**
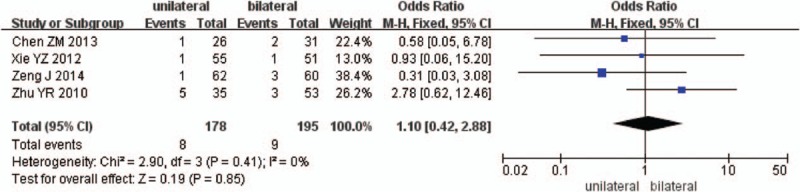
The standardized mean odds ratio (OR) estimate for nerve injury.

Four studies reported the nerve injury between unilateral and bilateral pedicle screw fixation with PLIF (n = 186 in the unilateral group, and 204 in the bilateral group). Meta-analysis showed unilateral group was similar compared with bilateral group in nerve injury. (*P* = .85, OR = 1.10 [0.42, 2.88]; heterogeneity: *P* = .41, *I*^2^ = 0%, fixed-effect model, Fig. 14).

**Figure 15:**
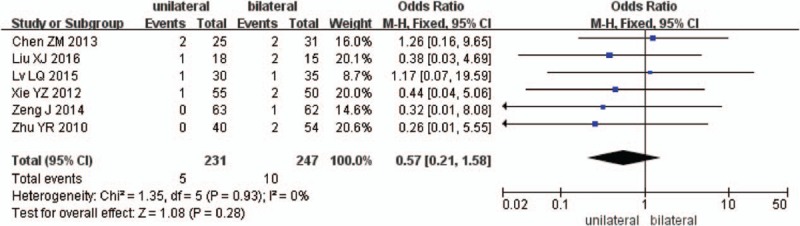
The standardized mean odds ratio (OR) estimate for dural injury.

Six studies reported the dural injury between unilateral and bilateral pedicle screw fixation with PLIF (n = 232 in the unilateral group, and 268 in the bilateral group). Meta-analysis showed unilateral group was similar compared with bilateral group in dural injury. (*P* = .28, OR = 0.57 [0.21, 1.58]; heterogeneity: *P* = .93, *I*^2^ = 0%, fixed-effect model, Fig. 15).

### 3.7 Publication bias

Publication bias were detected by STATA 12.0, and no publication bias found for all included studies (all *P* > .05). The funnel plot did not indicate any publication bias in blood loss (Begg, *P* = .133; Egger, *P* = .146); operation time (Begg, *P* = .806; Egger, *P* = .69); length of hospital stay (Begg, *P* = .452; Egger, *P* = .689); JOA score before surgery (Begg, *P* = 1.000; Egger, *P* = .957); JOA score at final follow-up (Begg, *P* = 1.000; Egger, *P* = .925); ODI score before surgery (Begg, *P* = .221; Egger, *P* = .114); ODI score at final follow-up (Begg, *P* = .806; Egger, *P* = .746); VAS score before surgery (Begg, *P* = 1.000; Egger, *P* = .974); VAS score at final follow-up (Begg, *P* = .902; Egger, *P* = .875); fusion rate (Begg, *P* = .711; Egger, *P* = .947); total complications (Begg, *P* = .436; Egger, *P* = .206); infection (Begg, *P* = 1.000; Egger, *P* = .779); nerve injury (Begg, *P* = 1.000; Egger, *P* = .744); and dural injury (Begg, *P* = .089; Egger, *P* = .054).

## 4 Discussion

LDD is a common disease, which can lead to significant disability and severe pain. With the population aging trends and lifestyle changes of the elderly, LDD has become an important degenerative disease causing spinal dysfunction and seriously affect the work and life of patients. To achieve favorable clinical results, adequate decompression around the spinal cord and nerve roots with or without fusion is essential. Bilateral pedicle screw fixations has been regarded as the standard surgical procedure in spinal fusion surgery to provide rigid lumbar spinal fixation.^[9,27,28]^ But this procedure has also been suspected to result in adjacent segments degeneration, device-related osteoporosis, and higher risk of implant-related complications.

Recently, unilateral pedicle screw fixation has been reported to be as effective as bilateral fixation in lumbar fusion.^[14,29]^ Hu et al^[30]^ conducted a systematic review comparing unilateral versus bilateral pedicle screw fixation in TLIF, Wang et al^[31]^ conducted a meta-analysis comparing unilateral versus bilateral pedicle screw fixation of minimally invasive TLIF. But they did not compare the unilateral with bilateral pedicle screw fixation in PLIF. Zhong et al^[32]^ performed a meta-analysis comparing unilateral and bilateral fixation with PLIF, but the JOA scores and the incidence rate of infection, nerve injury and dural injury were not analyzed. And Zhong study showed that the final follow-up VAS score was significantly different between unilateral and bilateral fixation, while in this paper, there were no statistical differences. That is probably due to the number of papers included was too small.^[32]^ Therefore, we conducted this meta-analysis to analyze the data of the unilateral and bilateral pedicle screw fixation in PLIF.

Although no randomized controlled trials (RCTs) included in this meta-analysis, we considered the included studies suitable for meta-analysis because of the high quality of those included studies according to the NOQAS, and the baseline variables were similar. Surgical trauma (including blood loss and operation time), clinical outcomes (including JOA, ODI, VAS score, and the length of hospital stay), fusion rate, and complications (including total complications, infection, dural injury, and nerve injury) were analyzed in this study.

Surgical trauma were always assessed by operation time and blood loss.^[33]^ This meta-analysis showed that unilateral pedicle screw fixation has better results in both the blood loss and the operation time, indicated that the surgical trauma is smaller in unilateral pedicle screw fixation compared with bilateral. This results may be attribute to only one side of muscle was dissected and unilateral pedicle screw placed.^[34,35]^

The clinical outcomes after lumber surgery were often evaluated using JOA, VAS, ODI scores, and the length of hospital stay. The pooled data showed that there were no significant difference in preoperative and final follow-up JOA scores, preoperative and final follow-up VAS scores, and preoperative ODI scores. But statistically significant in final follow-up ODI scores and the length of hospital stay. Both 2 methods could achieved sufficient improvement in JOA, VAS, ODI scores, hence, both methods were effective in decompression of spinal cord and nerve improvement. But unilateral fixation may superior to bilateral in spinal function recovery regarding the final follow-up ODI scores and the length of hospital stay.

We selected total complications, infection, nerve injury, and dural injury to evaluate postoperative complications. Both side of muscle dissection and bilateral pedicle screw placement in bilateral group, resulting in the increased chances of a surgical infection and nerve injury.^[18]^ However, the pooled data showed no significant difference in the total complications, infection, dural injury, and nerve injury between 2 groups.

We also analyzed the fusion rate between unilateral and bilateral pedicle screw fixation. This study found that there was no significant difference between the 2 groups in fusion rate. Fusion could greatly improve the stiffness of internal fixation.^[36]^ Fusion is important for unilateral pedicle screw fixation particularly, so the completely resection of intervertebral disc and the correctly scraping of cartilage endplates are significant in surgery.

There are several limitations of this study. First, there was no RCTs included this study, we need RCTs for further study. Second, adjacent level degeneration after lumbar interbody fusion is an important radiographic evaluation of surgical outcome, this result was not analyzed. Third, the sample sizes of these studies might not be large enough to show significant differences between the 2 groups, more studies should be included to improve the power of the findings. Fourth, the length of follow-up varied among the included studies, and thus may influence our results. Finally, we only included the studies in English and Chinese, and some relevant studies reported in other languages were not included due to a language limitation.

## 5 Conclusions

In summary, the findings of this meta-analysis suggest that both unilateral pedicle screw fixation with PLIF and bilateral pedicle screw fixation with PLIF for LDD have effective results in clinical outcomes. Both 2 methods may result in similar outcomes of fusion rate and complications. However, unilateral group produced more satisfactory efficacy in the blood loss and the operation time. Further studies with high methodological quality and long-term follow-up periods are needed to evaluate the 2 procedures for LDD treatment.
